# Association of parental obesity with cardiometabolic risk factors in their children: The CASPIAN-V study

**DOI:** 10.1371/journal.pone.0193978

**Published:** 2018-04-11

**Authors:** Hanieh-Sadat Ejtahed, Ramin Heshmat, Mohammad Esmaeil Motlagh, Shirin Hasani-Ranjbar, Hasan Ziaodini, Majzoubeh Taheri, Zeinab Ahadi, Tahereh Aminaee, Gita Shafiee, Azam Goodarzi, Mostafa Qorbani, Roya Kelishadi

**Affiliations:** 1 Obesity and Eating Habits Research Center, Endocrinology and Metabolism Molecular -Cellular Sciences Institute, Tehran University of Medical Sciences, Tehran, Iran; 2 Chronic Diseases Research Center, Endocrinology and Metabolism Population Sciences Institute, Tehran University of Medical Sciences, Tehran, Iran; 3 Department of Pediatrics, Ahvaz Jundishapur University of Medical Sciences, Ahvaz, Iran; 4 Endocrinology and Metabolism Research Center, Endocrinology and Metabolism Clinical Sciences Institute, Tehran University of Medical Sciences, Tehran, Iran; 5 Health Psychology Research Center, Education Ministry, Tehran, Iran; 6 Office of Adolescents and School Health, Ministry of Health and Medical Education, Tehran, Iran; 7 Medical Faculty, Tarbiat Modarres University, Tehran, Iran; 8 Non-communicable Diseases Research Center, Alborz University of Medical Sciences, Karaj, Iran; 9 Child Department of Pediatrics, Child Growth and Development Research Center, Research Institute for Primordial Prevention of Non-communicable Disease, Isfahan University of Medical Sciences, Isfahan, Iran; Beijing Key Laboratory of Diabetes Prevention and Research, CHINA

## Abstract

**Background:**

The family environment has a crucial role in the development of childhood obesity and related cardiometabolic disorders. This study aims to investigate the association of parental obesity and cardiometabolic risk factors in their children.

**Methods:**

This multicentric cross-sectional study was performed on 14400 students (aged 7–18 years) and one of their parents. Students were recruited by multistage, stratified cluster sampling from urban and rural areas of 30 provinces of Iran. Fasting venous blood was obtained from a random sample of 4200 students. Demographic, anthropometric and clinical variables were collected.

**Results:**

Data of 14002 students and results of blood samples of 3483 of them were complete and included in the current study. The prevalence of obesity in children, fathers, and mothers was 11.4%, 10.6%, and 24.2%, respectively. In students, the most commonly observed metabolic abnormality was low HDL-C (29.5%); the prevalence of metabolic syndrome and dyslipidemia was 5% and 55.7%, respectively. Significant correlations were observed between the body mass index (BMI) and waist circumference of parents and weight, height, BMI, and waist circumference, as well as systolic and diastolic blood pressure (BP) of their children (*P*< 0.05). In the multivariate model, the risk of excess weight (OR: 1.30, 95%CI: 1.17–1.44), obesity (OR: 1.36, 95%CI: 1.18–1.59), abdominal obesity (OR: 1.16, 95%CI: 1.05–1.29) and elevated BP (OR: 1.17, 95%CI: 1.04–1.31) were higher in those students whose parents had excess weight compared with other students. Parental obesity did not have significant association with metabolic syndrome and dyslipidemia in their children.

**Conclusions:**

Parental history of obesity could be used as a practical approach for the early preventive measures and identification of children at risk of cardiometabolic complications.

## Introduction

Increasing prevalence of childhood obesity and related cardiometabolic complications has made it a public health challenge [1]. I In 50 to 60% of cases, childhood overweight and obesity would persist into adulthood, and in turn, it could increase the risk of morbidity and mortality in young adulthood [2, 3]. It is well established that pediatric obesity and metabolic syndrome increase the risk of most non-communicable diseases including cardiovascular diseases, type 2 diabetes and some cancers in adulthood [4]. Therefore, prevention of childhood excess weight is crucial for a good health status in adulthood.

Unhealthy behaviors are considered as risk factors for obesity; it indicates the important role of similar family lifestyle habits and environments [3]. As parents have crucial roles in constructing their children’s health behaviors, their weight status could be an important factor in this regard [5, 6]. Evidence from some Western countries has shown that parental obesity is associated with childhood obesity [7–9]. Fewer studies have investigated the relationships between parental weight status and metabolic complications in children, and whether children with parental obesity are at higher risk for cardimetabolic impairment remains to be determined [10].

According to the national school-based surveillance program in Iran, the prevalence of obesity increased from 4.79% in 2008 to 11.89% in 2011–2012 [1, 11]. Moreover, it is shown that overweight and/or obesity in Iranian students was significantly associated with parental overweight and/or obesity [12]. These evidences highlight the needs of considerable attention to prevention and control of excess weight from childhood. Because of differences in socio-cultural factors between countries, this study was conducted in a nationally representative sample of Iranian students to examine the association of parental weight status with obesity and cardiometabolic complications of their children.

## Materials and methods

### Study design and population

This cross-sectional multicentric study was conducted in 2015, as the fifth survey of a national school-based surveillance programin Iran, entitled “Childhood and Adolescence Surveillance and PreventIon of Adult Non-communicable Disease (CASPIAN-V)” study. Participants were 14400 students, aged 7–18 years, with one of their parents who were selected via multistage, stratified cluster sampling method from urban and rural areas of 30 provinces of Iran. 480 students were selected from each province according to the living area (urban/rural) and school level, proportional to size with equal sex ratio. A subsample of 4200 students were randomly selected for biochemical test [13].

In this study, participants were recruited voluntarily. The study protocol and consent procedure were approved by the Research and Ethics Council of Isfahan University of Medical Sciences (Project code: 194049). After explaining the study objectives to participants, verbal consent was obtained from students; parents gave written informed consents on behalf of their children as well as themselves.

### Data collection

Trained health care professional teams completed two valid and reliable questionnaires related to health status and health-related behaviors of children and parents [14, 15]. Participation of one of the parents to their choice was necessary. The student’s questionnaire included questions about body size, life satisfaction, dietary habits, health behaviors, physical activity, leisure time activities, and tobacco use; the parents’ questionnaire consisted of questions about family characteristics, family history of chronic diseases, dietary habits, leisure time activities, parents’ sleep pattern, and parents’ anthropometric measures.

Screen time was calculated as the time (hours/day) spent watching television, working with personal computer and playing electronic games. Physical activity was assessed by asking about the frequency of their leisure time physical activity during the week. Having at least 30 minutes daily exercise which led to heavy sweating or increased heart rate was considered as physical activity. Socioeconomic status (SES) was calculated using principle component analysis considering variables including parental education, parents’ job, possessing private car, school type (public/private), and having personal computer in home. Eating habits were evaluated by asking some question about healthy eating behaviors including the consumption frequency of breakfast, fruit, vegetables, and milk and unhealthy eating behaviors including the consumption frequency of sugar-sweetened beverages (carbonated drinks and artificial fruit juices), fast foods (sausages, hamburgers and pizza), sweets (cakes, candies, biscuits, and chocolates) and salty snacks (chips, pretzels, and salty puff pates). Healthy and unhealthy dietary habits were defined using principle component analysis.

### Clinical and laboratory measurements

Trained health care experts performed anthropometric measurements of both students and one of their parents under standard protocols and by using calibrated instruments. Weight was measured to the nearest 100 g while the participants were minimally clothed, without shoes. Height was measured to the nearest 0.1 cm in a standing position, without shoes [16]. Body mass index (BMI) was calculated by dividing weight (kg) to height squared (m^2^). The growth charts of the World Health Organization were used to categorize BMI in children [17]. Waist circumference (WC) was measured to the nearest 0.1 cm, using a tape meter, at the umbilical level over light clothing. Blood pressure (BP) was measured twice in the sitting position on the right arm after at least 5 minutes of rest, using mercury sphygmomanometers. The mean of the two measurements was considered as the participant’s BP.

Venous blood samples were collected from students after 12-h- overnight fasting. Fasting blood glucose (FBG), triglycerides (TG), total cholesterol (TC), high-density lipoprotein-cholesterol (HDL-C), and low- density lipoprotein-cholesterol (LDL-C) were measured enzymatically by Hitachi auto-analyzer (Tokyo, Japan) [18, 19,20].

### Definitions of study’s criteria

Parental excess weight and obesity were defined as having BMI≥25 kg/m^2^ and BMI≥30 kg/m^2^, respectively. The definition of parental abdominal obesity was considered as WC ≥95 cm [21].

Metabolic syndrome in children was defined according to the Adult Treatment Panel III definition modified for children and adolescents, as having at least 3 of the following abnormalities: 1) abdominal obesity (WC≥ 90^th^ percentile); 2) low HDL-C (HDL< 40 mg/dL except in15–19-year-old boys, in whom the cut-off was < 45 mg/dL); 3) elevated TG (TG>100 mg/dL); 4) elevated FBG (FBG> 100 mg/dL); 5) elevated systolic or diastolic BP (SBP or DBP> 90^th^ percentile adjusted by age, sex, and height) [22].

Excess weight and obesity in children were considered as age-and gender-specific BMI greater than 85th percentile and BMI greater than 95th percentile, respectively [11]. Dyslipidemia in children was defined according to the recent recommendation of the American Heart Association as at least the abnormality of one of the components of lipid profile (TG, HDL-C, LDL-C, TC): elevated LDL-C: LDL-C> 110 mg/dL; elevated TC: TC> 200 mg/dL; Low HDL-C: HDL< 40 mg/dL except in 15–19- year- old boys, in whom the cut-off was < 45 mg/dL; elevated TG: TG>100 mg/dL [23].

### Statistical analysis

Continuous and categorical variables are presented as mean (standard deviation, SD) and number (percentage), respectively. Statistical analyses were conducted using STATA version 11.0 (STATA Statistical Software: Release 11. StataCorp LP. Package, College Station, TX, USA) (S1 File). Clinical and biochemical characteristics of children were compared according to the parental weight status by independent t-test. Prevalence of cardiovascular risk factors and components of metabolic syndrome in children were compared according to the parental weight status using Chi-square test. Correlations between parental anthropometric indices and children clinical characteristics were determined using Pearson correlation coefficient. Multiple logistic regression analysis was used to examine the association between parental weight status and children’s metabolic syndrome and cardiovascular risk factors adjusted for age of children, sex of children and parents, residential area, parental socio-economic status, screen time, and physical activity, dietary habits, and BMI of children. Data are presented as odds ratio (OR) with 95% confidence interval (CI). Goodness of fit (GOF) of logistic regression models was assessed by considering the survey sampling design and using the F-adjusted mean GOF residual. *P*value of more than 0.05 showed good fitness of the model.

## Results

In this national study, 14002 students (50.6% boys and 49.4% girls) with a mean age of 12.3y and one of their parents (2569 fathers and 11433 mothers) completed the survey, and 3843 blood samples were available. Most children (71.4%) were from urban area and 58.8% of them studied in elementary school (grades 1–6). Demographic characteristics of children and their parents are presented in Table 1. It shows that the mean BMI of fathers and mothers were 25.1kg/m^2^and 26.7 kg/m^2^, respectively. The prevalence of obesity was 11.4% (10% in girls and 12% in boys) in students, and 10.6%, and 24.2% in their fathers and mothers, respectively. In children, the most common metabolic abnormality was low HDL-C (29.5%), and the prevalence of metabolic syndrome and dyslipidemia was 5% and 55.7%, respectively.

**Table 1:** Demographic characteristics of parents and children: The CASPIAN-V study.

	Fathers	Mothers	Children
Age (y)	44.2±7.1	38.1±6.5	12.3±3.1
Weight (kg)	73.7±13.2	67.3±12.9	41.4±17.1
Height (cm)	170.2±15.2	158.6±13.1	146.6±17.5
BMI (kg/m^2^)	25.1±4.1	26.7±5.0	18.5±4.7
WC (cm)	87.0±16.6	87.7±14.3	66.7±12.2
Excessweight	1343 (52.3)	7011 (61.3)	2945 (20.8)
Obesity	272 (10.6)	2766 (24.2)	1615 (11.4)
Abdominal obesity	893 (35.3)	3472 (30.4)	2972 (21.1)

Data are presented as mean±SD or number(percentage).

BMI, body mass index; WC, waist circumference.

Parental excess weight: BMI≥25 kg/m^2^; Parental obesity: BMI≥30 kg/m^2^; Parental abdominal obesity: waist circumference≥95 cm; Children excess weight: BMI> 85^th^percentile; Children obesity: BMI≥ 95^th^ percentile; Children abdominal obesity: WC>90^th^percentile

Table 2 shows the clinical characteristics of children according to the parental weight status. The mean of weight, height, BMI, WC, SBP and DBP were significantly different between children whose parents were obese or not, had excess weight or not, and had abdominal obesity or not (*P*< 0.01). The prevalence of cardiovascular risk factors and metabolic syndrome components of children according to the parental weight status is presented in Table 3. Students with obesity and abdominal obesity in parents had significantly higher prevalence of obesity, abdominal obesity, excess weight, elevated BP and DBP (*P*< 0.05). In addition, children of parents with excess weight had significantly higher prevalence of obesity, abdominal obesity, excess weight, elevated BP and DBP (*P*< 0.05). The frequency of elevated LDL-C was significantly higher in students with abdominal obesity in their parents than in other students (19.4% vs. 16.7%, respectively, *P*< 0.05). No significant difference was seen in the prevalence of dyslipidemia in children according to the parental weight status. Metabolic syndrome in children with obese parents was more prevalent than in children with normal-weight parents (6.4% vs. 4.5%, respectively, *P*< 0.05).

**Table 2:** Means of clinical and biochemical characteristics of children according to the parental weight status: The CASPIAN-V study.

	Total	Parental excess weight		Parental obesity		Parental abdominal obesity	
		Yes	No	Yes	No	Yes	No
Age (y)	12.3±3.1	12.4±3.2	12.1±3.1*	12.4±3.2	12.2±3.2	12.5±3.2	12.2±3.1*
Weight (kg)	41.4±17.1	42.4±17.2	39.7±16.9*	43.6±18.1	40.7±16.8*	43.2±17.7	40.5±16.6*
Height (cm)	146.6±17.5	147.2±17.4	145.5±17.6*	147.2±17.2	146.3±17.6*	148.1±17.6	145.8±17.4*
BMI (kg/m^2^)	18.5±4.7	18.8±4.7	18.0±4.7*	19.3±5.1	18.3±4.6*	18.9±4.8	18.3±4.4*
WC (cm)	66.7±12.2	67.5±12.2	65.4±11.9*	68.4±13.0	66.2±11.9*	68.3±12.6	65.9±11.8*
FBS (mg/dl)	91.6±12.1	91.8±12.7	91.3±11.1	91.4±12.0	91.7±12.1	91.9±14.5	91.5±10.7
TC (mg/dl)	153.8±27.4	153.8±26.9	153.9±28.3	153.2±26.6	154.0±27.7	154.6±27.0	153.6±27.5
TG (mg/dl)	88.0±45.2	88.1±44.0	88.0±47.0	87.0±42.3	88.4±46.1	86.6±42.4	88.7±46.1
LDL-C (mg/dl)	90.0±22.6	89.8±22.3	90.3±23.2	89.7±22.7	90.1±22.7	90.6±22.6	89.8±22.5
HDL-C (mg/dl)	46.2±9.9	46.3±9.9	45.9±9.9	46.1±9.7	46.2±10.0	46.4±10.2	46.0±9.9
SBP (mmHg)	99.2±13.1	99.7±13.1	98.2±13.1*	100.2±12.6	98.8±13.2*	99.9±12.9	98.8±13.2*
DBP (mmHg)	63.8±10.4	64.1±10.5	63.3±10.3*	64.6±10.4	63.6±10.4*	64.3±10.7	63.6±10.4*

Data are presented as mean±SD.

BMI, body mass index; WC, waist circumference; FBS, fasting blood sugar; TC, total cholesterol; TG, triglycerides; LDL-C, low-density lipoprotein cholesterol; HDL-C, high-density lipoprotein cholesterol; SBP, systolic blood pressure; DBP, diastolic blood pressure.

Parental excess weight: BMI≥25 kg/m^2^; Parental obesity: BMI≥30 kg/m^2^; Parental abdominal obesity: waist circumference≥95 cm.

* Significant difference of children clinical characteristics according to the parental weight status (*P*< 0.01)

**Table 3:** Prevalence of cardiovascular risk factors and metabolic syndrome components in children according to the parental weight status: The CASPIAN-V study.

	Parental excess weight		Parental obesity		Parental abdominal obesity	
	Yes	No	Yes	No	Yes	No
Excess weight	1899(22.9)	975(17.4)*	789(26.2)	2085(19.2)*	988(22.8)	1883(19.8)*
Obesity	1079(13.0)	497(8.9)*	476(15.8)	1100(10.1)*	575(13.3)	993(10.4)*
Abdominal obesity	1860(22.4)	1041(18.6)*	771(25.6)	2130(19.6)*	1030(23.8)	1873(19.7)*
Elevated FBG	92(4.2)	66(4.2)	33(4.0)	125(4.2)	51(4.3)	103(4.0)
Elevated TG	609(27.8)	436(27.4)	225(27.1)	820(27.7)	313(26.2)	738(28.5)
Elevated TC	104(4.7)	84(5.3)	29(3.5)	159(5.4)*	56(4.7)	129(5.0)
Elevated LDL	381(17.4)	284(17.9)	139(16.8)	526(17.8)	232(19.4)	433(16.7)*
Low HDL	261(31.5)	856(29.0)	642(29.3)	475(29.9)	361(30.3)	754(29.2)
Elevated SBP	277(3.4)	150(2.7)*	92(3.1)	335(3.1)	130(3.0)	297(3.1)
Elevated DBP	893(10.9)	516(9.3)*	355(11.9)	1054(9.8)*	487(11.4)	936(9.9)*
Elevated blood pressure	986(12.0)	574(10.4)*	383(12.9)	1177(10.9)*	535(12.5)	1037(11.0)*
Dyslipidemia	1222(55.7)	886(55.7)	462(55.7)	1646(55.7)	684(57.3)	1426(55.1)
Metabolic syndrome	115(5.4)	66(4.3)	51(6.4)	130(4.5)*	57(4.9)	128(5.1)

Data are presented as the number of subjects (percentages).

FBG, fasting blood glucose; TC, total cholesterol; TG, triglycerides; LDL-C, low-density lipoprotein cholesterol; HDL-C, high-density lipoprotein cholesterol; SBP, systolic blood pressure; DBP, diastolic blood pressure.

Parental excess weight: BMI≥25 kg/m^2^; Parental obesity: BMI≥30 kg/m^2^; Parental abdominal obesity: waist circumference≥95 cm; Dyslipidemia: at least one of the components (TG, HDL, LDL, TC) are abnormal; Excess weight: BMI> 85^th^percentile; Obesity: BMI > 95th; Abdominal obesity: WC> 90^th^ percentile; Low HDL: HDL< 40 mg/dL (except in boys 15–19 y old, that cut-off was < 45 mg/dL); High LDL: LDL> 110 mg/dL; High TG: TG>100 mg/dL; High TC: TC> 200 mg/dL; High FBS: FBS > 100 mg/dL; High blood pressure: BP> 90^th^ (adjusted by age, sex, height); Metabolic syndrome: ATP-III criteria.

* Significant difference in the prevalence of cardiovascular risk factors and metabolic syndrome components in children according to the parental weight status (*P*< 0.05).

Table 4 presents the correlations between parental anthropometric indices and children clinical characteristics. Significant correlations were observed between BMI and WC of parents with weight, height, BMI, WC, SBP, and DBP in their children (*P*< 0.05). Correlation between children’s HDL-C and parental WC was seen only in father-child pairs (*P*< 0.05). Logistic regression models were applied on the association between metabolic syndrome and cardiovascular risk factors in children and parental weight status (Table 5). Students with obese and abdominal obese parents had significantly higher odds of being excess weight and having obesity, abdominal obesity and elevated BP. Moreover, the odds of excess weight, obesity, abdominal obesity and elevated BP were higher in students whose parents had excess weight compared with children of normal-weight parents (*P*< 0.05). Theses associations remained significant after adjusting for age of children, sex of children and parents, living area, parental socio-economic status, screen time, physical activity, dietary habits and BMI of children (*P*< 0.05). The odds of elevated LDL-C was 31% higher in children with abdominal obesity in parents compared with other children. Significant association was documented between parental obesity and children metabolic syndrome, but it was no more significant after adjusting for covariates. The fitness of all models was good (*P* > 0.05).

**Table 4:** Correlations between parental anthropometric indices and children clinical characteristics: The CASPIAN-V study.

	Fathers		Mothers		Parents	
	BMI	WC	BMI	WC	BMI	WC
Weight	0.12*	0.08*	0.11*	0.08*	0.10*	0.08*
Height	0.065*	0.072*	0.06*	0.078	0.06*	0.07*
BMI	0.115*	0.06*	0.12*	0.075*	0.11*	0.07*
WC	0.13*	0.13*	0.1*	0.1*	0.10*	0.10*
FBG	-0.013	0.001	0.01	0.03	0.01	0.025
TC	0.004	0.05	-0.001	-0.01	0.001	0.003
TG	-0.003	0.027	-0.007	-0.03	-0.007	-0.017
LDL	-0.006	0.018	-0.003	-0.006	-0.004	0.001
HDL	0.03	0.078*	0.01	0.01	0.01	0.02
SBP	0.043*	0.049*	0.07*	0.05*	0.06*	0.05*
DBP	0.049*	0.061*	0.05*	0.03*	0.045*	0.04*

Data are presented as the correlation coefficient.

BMI, body mass index; WC, waist circumference; FBG, fasting blood glucose; TC, total cholesterol; TG, triglycerides; LDL-C, low-density lipoprotein cholesterol; HDL-C, high-density lipoprotein cholesterol; SBP, systolic blood pressure; DBP, diastolic blood pressure.

* Significant correlations between parental anthropometric indices and children clinical characteristics (*P*< 0.05).

**Table 5:** Odds ratio and 95% confidence interval of metabolic syndrome and cardiovascular risk factors in children associated with parental weight status: The CASPIAN-V study.

	Parental excess weight	Parental obesity	Parental abdominal obesity
Excess weight			
Model 1	1.40(1.29–1.53)*	1.49(1.36–1.64)*	1.19(1.09–1.31)*
Model 2	1.30(1.17–1.44)*	1.46(1.29–1.65)*	1.10(0.98–1.23)
F-adjusted GOF (*P*)	0.82(0.60)	0.19(0.99)	0.95(0.49)
Obesity			
Model 1	1.53(1.37–1.71)*	1.67(1.48–1.87)*	1.31(1.18–1.47)*
Model 2	1.36(1.18–1.59)*	1.60(1.35–1.90)*	1.22(1.04–1.43)*
F-adjusted GOF (*P*)	1.99(0.06)	0.77(0.64)	2.01(0.06)
Abdominal obesity			
Model 1	1.26(1.16–1.38)*	1.41(1.28–1.55)*	1.27(1.17–1.39)*
Model 2	1.16(1.05–1.29)*	1.32(1.16–1.50)*	1.09(0.97–1.22)
F-adjusted GOF (*P*)	0.52(0.85)	1.20(0.32)	0.70(0.70)
Elevated FBG			
Model 1	1.01(0.73–1.40)	0.94(0.63–1.39)	1.07(0.76–1.52)
Model 2	0.93(0.65–1.33)	0.96(0.54–1.69)	1.10(0.72–1.68)
F-adjusted GOF (*P*)	2.22(0.051)	2.36(0.052)	0.66(0.73)
Model 3	0.92(0.64–1.33)	0.93(0.53–1.66)	1.09(0.71–1.67)
F-adjusted GOF (*P*)	1.26(0.3)	1.75(0.14)	2.03(0.06)
Elevated TG			
Model 1	1.02(0.88–1.17)	0.97(0.82–1.15)	0.89(0.76–1.04)
Model 2	0.96(0.78–1.17)	0.86(0.65–1.15)	0.83(0.67–1.01)
F-adjusted GOF (*P*)	1.98(0.06)	1.14(0.38)	0.33(0.95)
Model 3	0.95(0.77–1.17)	0.86(0.64–1.15)	0.83(0.67–1.02)
F-adjusted GOF (*P*)	1.90(0.06)	1.38(0.26)	0.25(0.98)
Elevated TC			
Model 1	0.89(0.66–1.20)	0.64(0.43–0.95)*	0.94(0.68–1.29)
Model 2	0.79(0.47–1.32)	0.67(0.35–1.27)	1.07(0.71–1.61)
F-adjusted GOF (*P*)	0.82(0.60)	2.30(0.052)	2.34(0.054)
Model 3	0.79(0.48–1.33)	0.68(0.36–1.41)	1.08(0.72–1.63)
F-adjusted GOF (*P*)	0.20(0.99)	0.55(0.82)	1.47(0.22)
Elevated LDL-C			
Model 1	0.97(0.82–1.14)	0.93(0.76–1.14)	1.20(1.01–1.43)*
Model 2	1.02(0.78–1.33)	0.97(0.70–1.36)	1.31(1.01–1.72)*
F-adjusted GOF (*P*)	1.17(0.36)	0.82(0.60)	2.02(0.06)
Model 3	1.02(0.78–1.33)	0.98(0.70–1.37)	1.31(0.99–1.73)
F-adjusted GOF (*P*)	1.09(0.41)	0.43(0.90)	2.12(0.05)
Low HDL-C			
Model 1	0.97(0.84–1.12)	1.13(0.95–1.33)	1.05(0.91–1.22)
Model 2	0.88(0.73–1.06)	1.02(0.82–1.25)	0.91(0.72–1.15)
F-adjusted GOF (*P*)	2.80(0.052)	2.90(0.05)	3.10(0.051)
Model 3	0.87(0.72–1.05)	1.0(0.81–1.25)	0.91(0.71–1.15)
F-adjusted GOF (*P*)	2.10(0.052)	1.90(0.06)	2.05(0.051)
Dyslipidemia			
Model 1	0.99(0.88–1.14)	1.01(0.86–1.17)	1.09(0.95–1.25)
Model 2	0.98(0.84–1.13)	0.95(0.81–1.12)	1.02(0.86–1.22)
F-adjusted GOF (*P*)	0.73(0.68)	1.39(0.25)	1.80(0.13)
Model 3	0.97(0.83–1.12)	0.94(0.80–1.11)	1.02(0.85–1.21)
F-adjusted GOF (*P*)	2.22(0.06)	2.10(0.05)	0.64(0.75)
Elevated blood pressure			
Model 1	1.18(1.06–1.32)*	1.20(1.06–1.36)*	1.16(1.04–1.29)*
Model 2	1.22(1.08–1.37)*	1.26(1.08–1.46)*	1.21(1.05–1.39)*
F-adjusted GOF (*P*)	0.71(0.69)	0.99(0.46)	1.31(0.26)
Model 3	1.17(1.04–1.31)*	1.17(1.01–1.37)*	1.17(1.02–1.35)*
F-adjusted GOF (*P*)	1.60(0.14)	1.26(0.28)	1.28(0.28)
Metabolic syndrome			
Model 1	1.27(0.93–1.73)	1.43(1.03–2.01)*	0.96(0.69–1.32)
Model 2	1.16(0.82–1.65)	1.09(0.63–1.89)	0.70(0.41–1.19)
F-adjusted GOF (*P*)	0.41(0.91)	2.25(0.05)	1.05(0.44)
Model 3	1.10(0.78–1.56)	0.99(0.57–1.73)	0.65(0.37–1.11)
F-adjusted GOF (*P*)	2.11(0.05)	2.25(0.056)	1.90(0.11)

Data are presented as the odds ratio (95% confidence interval).

FBG, fasting blood glucose; TC, total cholesterol; TG, triglycerides; LDL-C, low-density lipoprotein cholesterol; HDL-C, high-density lipoprotein cholesterol; GOF, goodness of fit.

Parental excess weight: BMI≥25 kg/m^2^; Parental obesity: BMI≥30 kg/m^2^; Parental abdominal obesity: waist circumference≥95 cm; Dyslipidemia: at least one of the components (TG, HDL, LDL, TC) are abnormal; Excess weight: BMI> 85th; Obesity: BMI > 95th; Abdominal obesity: WC> 90th; Low HDL: HDL< 40 mg/dL (except in boys 15–19 y old, that cut-off was < 45 mg/dL); High LDL: LDL> 110 mg/dL; High TG: TG>100 mg/dL; High TC: TC> 200 mg/dL; High FBS: FBS > 100 mg/dL; High blood pressure: BP> 90th (adjusted by age, sex, height); Metabolic syndrome: ATP-III criteria, modified for pediatric age.

Model 1: Crude model.

Model 2: Adjusted model for age of children, sex of children and parents, living area, parental socio-economic status, dietary habits, screen time, and physical activity.

Model 3: Extra adjusted for BMI of children.

* Significant association(*P*< 0.05).

## Discussion

In this multicentric study, the association of parental excess weight with cardiometabolic risk factors in their children was investigated. We found significant positive association between excess weight in parents and in their children. Children with obese and abdominal obese parents had significantly higher odds of having excess weight, obesity, abdominal obesity and elevated BP. These results are consistent with some previous studies [8, 9, 12, 24–26]. A recent study on a large international sample from 12 countries reported positive associations between parental and child overweight as well as between parental education and child overweight. Relation between maternal and child overweight was seen in all countries except Kenya and association between paternal and child overweight was found in seven countries [27]. In another recent study, 1521 Sicilian children were studied to identify the parent-related risk factors for childhood obesity and to find children who need early intervention strategies. They showed that the OR of childhood obesity was more than two times higher in children whose mothers were overweight or obese. Excess weight in fathers was also a risk factor for childhood obesity [28].

Although we did not analyze the relationship between parental and child weight status separately for mothers and fathers, some of previous studies reported that maternal adiposity correlated more strongly with children weight status than paternal adiposity [26, 27, 29]. However, it is shown that correlation between overweight/obese children-father pairs (odds ratio: 3.2, 95% CI: 1.5–6.8) was stronger than that of overweight/obese children mother pairs (odds ratio: 2.2, 95% CI: 1.2–3.9). Moreover, significant correlations was reported between the BMI of children withthe BMI of fathersand mothers [24]. A recent study revealed that weight status of girls was affected equally with the weight of both parents, while boys were more affected by their fathers’ weight[30]. Inconsistencies observed in the effect size of these associations across different studies could attribute to variations in the study design, sampling methods, and socio-demographic factors. The family SES is associated with childhood obesity [31]. Moreover, it is shown that adolescents who had good relationships with their parents were less likely to engage in unhealthful weight-related behaviors [30]. Therefore, it seems that different SES and culture of countries would affect these associations.

In the current study, significant association was documented between parental obesity and children metabolic syndrome; however it did not remain significant after adjusting for covariates. This indicates the importance of the mediating effects of the life-style behaviors including screen time and physical activity. This observation suggests that parental obesity is likely to affect the cardiometabolic risk factors of children by influencing the physical activity habits and screen time of the family members. However, in the current study two components of metabolic syndrome in children, including abdominal obesity and elevated BP, were significantly associated with parental obesity. Therefore, our findings propose that children with obese parents are at increased risk of metabolic syndrome. A previous study showed that adolescents whose parents were overweight/obese had higher prevalence rate of metabolic syndrome. It also documented the mediating role of children’s obesity on the association between parental obesity and children’s metabolic syndrome [10]. This finding highlights the effect of childhood obesity on increasing risk of cardiometabolic complications in children. Therefore, family-based lifestyle interventions are needed for preventing obesity and its related cardiometabolic impairment.

Although in the current study, the prevalence of dyslipidemia was high among children, but there was no significant association between parental excess weight and dyslipidemia in their children. However, the odds ratio of having elevated LDL-C was significantly higher in children whose parents had abdominal obesity. In the present study, stronger association was seen between parental obesity and children weight status compared with parental obesity and children metabolic syndrome and dyslipidemia, suggesting that effects of genetic and environmental factors on weight status were more prominent than their effects on metabolic complications.

Our findings on significant correlations between parental BMI and WC with children’s weight, height, BMI, WC, SBP and DBP are consistent with some previous studies [29, 32, 33]. A previous study reported that maternal WC was strongly correlated with adolescent’s BMI, WC, and serum TG, as well as SBP and DBP[29]. In the present study, the correlation between children’s HDL-C and parental WC was seen only in father-child pairs indicating that children of obese fathers should be targeted for primary prevention of cardiometabolic impairment. The underlying mechanism could be genetic background and similarities in lifestyle factors in family members. Familial patterns of obesity could be partly explained by common dietary habits, physical activity levels and lifestyle in families [5, 6, 34, 35]. In addition, these patterns may be consequences of inappropriate social perceptions on healthiness of a chubby child.

In the present survey, large representative samples of students and their parents were studied. Therefore, the findings of this study can be generalized to some other populations. The other strength of this study is that our anthropometric data were based on physical examination, and not based on self-reported measures. Moreover, the data were obtained with high quality control. Therefore, reporting bias should be limited; even if it existed, the associations should be biased toward the null. One of the study limitations is that we could not determine the pubertal stage of students. Thus, the effect of hormonal changes on body composition and metabolic status during puberty could not be assessed. Moreover, because of the cross-sectional design of this study, no causality can be drawn between variables studied. At last, the parental blood samples were not collected and we could not evaluate the association of metabolic complications between children and their parents.

## Conclusion

The present study showed significant associations between general- and abdominal obesity in parents with excess weight, obesity, abdominal obesity and elevated BP in their children. Moreover, the risk of elevated LDL-C was significantly higher in children whose parents had abdominal obesity. Therefore, parental history of obesity could be used as an informative practical approach for the early identification of children at risk of cardiometabolic complications. Moreover, high-risk approach should be considered for prevention and control of obesity and related metabolic impairment in children with obese parents.

## Supporting information

S1 File Raw data underlying the findings of this study. (SAV)
